# Neuroprotective Effects of the Inert Gas Argon on Experimental Traumatic Brain Injury In Vivo with the Controlled Cortical Impact Model in Mice

**DOI:** 10.3390/biology11020158

**Published:** 2022-01-19

**Authors:** Fritz I. Schneider, Sandro M. Krieg, Ute Lindauer, Michael Stoffel, Yu-Mi Ryang

**Affiliations:** 1 Department of Neurosurgery, Klinikum Rechts der Isar, Technical University Munich, 81675 Munich, Germany; fritz.isaku.schneider@googlemail.com (F.I.S.); ulindauer@ukaachen.de (U.L.); michael.stoffel@helios-gesundheit.de (M.S.); Yu-Mi.Ryang@helios-gesundheit.de (Y.-M.R.); 2 Department of Neurosurgery, Klinikum der RWTH Aachen, RWTH Aachen University, 52062 Aachen, Germany; 3 Department of Neurosurgery, Helios Kliniken, 47805 Krefeld, Germany

**Keywords:** experimental TBI (traumatic brain injury), argon, inert gases, neurological outcome, brain contusion volume, brain edema volume, intracranial pressure (ICP), cerebral blood flow (CBF)

## Abstract

**Simple Summary:**

Traumatic brain injuries remain one of the leading causes of death in the western world and developing countries. There is an urgent need for causal therapies for such injuries. The noble gas argon has already shown promising results in in-vitro models. The influence of argon on the extent of damage after a craniocerebral trauma will be investigated in this study, in vivo, in mice. After the trauma, the animals were examined for neurological impairments and their brains were removed to detect brain edema and microscopically detectable alterations.

**Abstract:**

Argon has shown neuroprotective effects after traumatic brain injury (TBI) and cerebral ischemia in vitro and in focal cerebral ischemia in vivo. The purpose of this study is to show whether argon beneficially impacts brain contusion volume (BCV) as the primary outcome parameter, as well as secondary outcome parameters, such as brain edema, intracranial pressure (ICP), neurological outcome, and cerebral blood flow (CBF) in an in-vivo model. Subjects were randomly assigned to either argon treatment or room air. After applying controlled cortical impact (CCI) onto the dura with 8 m/s (displacement 1 mm, impact duration 150 ms), treatment was administered by a recovery chamber with 25%, 50%, or 75% argon and the rest being oxygen for 4 h after trauma. Two control groups received room air for 15 min and 24 h, respectively. Neurological testing and ICP measurements were performed 24 h after trauma, and brains were removed to measure secondary brain damage. The primary outcome parameter, BCV, and the secondary outcome parameter, brain edema, were not significantly reduced by argon treatment at any concentration. There was a highly significant decrease in ICP at 50% argon (*p* = 0.001), and significant neurological improvement (beamwalk missteps) at 25% and 50% argon (*p* = 0.01; *p* = 0.049 respectively) compared to control.

## 1. Introduction

Secondary brain damage emerges as a result of ischemia, excitotoxic events, brain edema, rising intracranial pressure (ICP), blood-brain barrier leakage, low systemic blood pressure, and hypoxia over the course of minutes to days after trauma [1,2]. Today’s therapeutic options focus on reducing the extent of secondary brain damage and thus improving neurological outcome. However, there is no established therapy that specifically targets preventing apoptosis of endangered cells in the penumbra [3].

Argon has shown several promising neuroprotective properties despite being chemically inert. Argon has shown significant reduction of neuronal cell death in vitro in mice after mechanical trauma and glucose/oxygen deprivation. Hippocampal preparations were traumatized with a constant kinetic energy pen and treated with argon for 72 h at concentrations of 25%, 50%, and 75%. The oxygen level was set at 21%. Propidium iodide (PI) staining demonstrated a significant reduction in secondary damage, which was most pronounced at a concentration of 50% argon [3,4]. Moreover, in-vivo studies showed protection against excitotoxic stress, i.e., NMDA-injections, and focal ischemia after middle cerebral artery occlusion (MCAO) in rats. In the case of NMDA injection, neuroprotectivity was demonstrated after one hour of treatment with 37.5% and 50% argon. In the MCAO experiments, a three-hour treatment with 50% argon significantly reduced cortical damage while increasing subcortical damage [4,5]. The mechanism of neuroprotection mediated by argon remains unclear. Recently, a whole string of possible candidates is being discussed involving hydrophobic interactions at protein cavities leading to altered gene expression via the ERK (extracellular signal-regulated kinase) 1/2 pathway and the modification of enzymes such as tissue plasminogen activator (tPA) [6,7,8,9]. In contrast, other studies have found an increase in inflammatory mediators after argon treatment following in-vivo ischemia in rats [10].

This study aims to examine argon in an in-vivo model of TBI (controlled cortical impact, CCI) and at determining the optimal length, concentration, and timing of posttraumatic argon treatment. The three applied argon concentrations are based on the results of an in-vitro TBI study [3]. Additionally, the systemic effects of argon were investigated.

The primary outcome parameter was brain contusion volume 24 h after TBI. Additionally, we measured intracranial pressure, neurological outcome, and brain water content 24 h after trauma as secondary outcome parameters.

## 2. Materials and Methods

### 2.1. Animals

Male C57Bl/6 mice (20–22 g) were obtained from a commercial breeder of laboratory animals (Charles River Laboratories, Sulzfeld, Germany). Animals were kept in Makrolon cages in a controlled environment (22.0 °C/55% humidity) and had free access to food and water before and during the experimental phase. Day/night cycles were kept constant with lighting from 8 a.m. to 8 p.m.

### 2.2. CCI

We used the widely established CCI [11,12]. For analgesia i.p. injections of buprenorphine were applied (0.1 mg per kg bodyweight; mg/kg BW). Animals were put in short isoflurane anesthesia (4–5%vol for induction, 1–2% to maintain) with 30% O_2_ and 70% N_2_O via facemask. Craniotomy of the right temporal bone was performed with preservation of the dura above the motor cortex. The size of the craniotomy was approximately 5 × 5 mm. CCI (Mouse-Katjuscha 2000, L. Kopacz, University of Mainz, Mainz, Germany) [13,14] was as follows: diameter of the impact tip, 3 mm; impact velocity, 8 m/s; duration, 150 ms. The bone flap was then replaced and affixed with histoacryl glue (Braun-Melsungen, Melsungen, Germany). The duration of the surgical procedure did not exceed 15 min.

### 2.3. Study Protocol

Blinding was performed by laboratory personnel who randomly presented the animals to the examiner for surgery and testing. Blinding was dissolved only after the complete assessment of the respective test series. Confounders were not controlled, as the procedure did not vary for each animal. For all test series, we have included a graphical flowchart to indicate the number of animals that were included in the experiments and the number included for analysis. Deaths of animals are reported in the result section. In several groups, some animals could not be included into analysis due to problems in methodology, mostly technical problems regarding the production of frozen sections and weighing the brain hemispheres to determine brain water content. The exclusions did not correlate to any specific treatment.

#### 2.3.1. Test Series A: Optimal Argon Concentration

**Figure 1:**
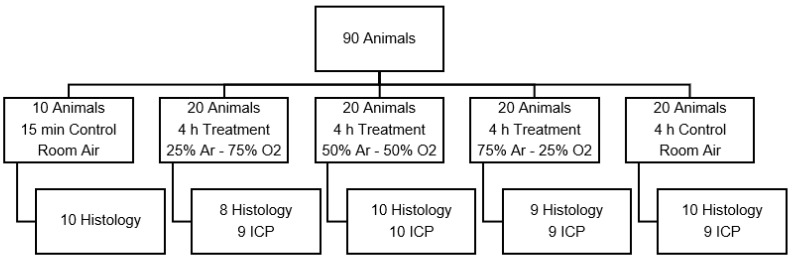
Animal distribution of test series A, which was designed to find the optimal argon concentration. (Ar: argon; O2: oxygen; ICP: intracranial pressure measurement).

Argon was administered at defined concentrations 30 min prior to trauma and continued for 4 h after trauma (Figure 1). The purpose of this test series was to measure neuroprotection under optimal circumstances, i.e., administering argon before trauma to ensure optimal brain tissue concentrations although these conditions are unlikely in a clinical setting. All argon was supplied by a commercial producer with a purity level above 99.9% (SWF, Sauerstoffwerk Friedrichshafen, Friedrichshafen, Germany). Medical grade oxygen, nitrogen, and nitrous oxide were readily available in our laboratory.

The Control group was exposed to room air for the entire duration of test series 1. All animals were placed in heated, semi-open boxes at a constant temperature of 26 degrees Celsius. For all test series, oxygen and argon levels were continuously monitored during treatment with an oxygen (Dräger Oxydig, Dräger, Lübeck, Germany) and argon meter (Servopro Monoexact, Servomex, Hamm, Deutschland). A continuous fresh gas flow of at least 0.1 L/min was established with a custom, calibrated flowmeter for oxygen, nitrogen, and argon, leading to a complete exchange of gas in the box within less than 15 min. The temperature in the boxes was fixed at 26 °C.

#### 2.3.2. Test Series B: Optimal Duration of Treatment

**Figure 2:**
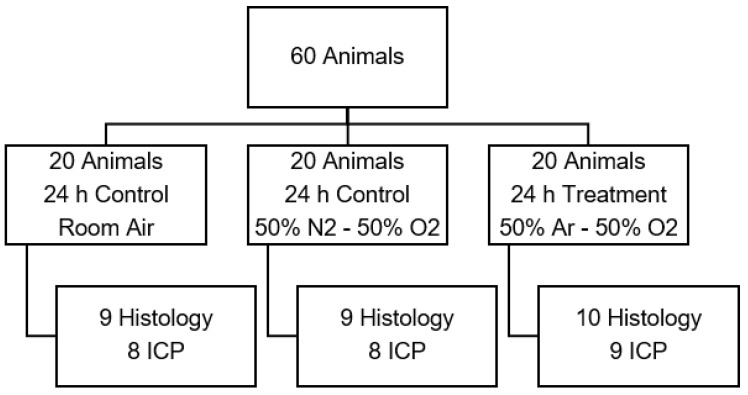
Animal distribution of test series B to examine the influence of argon mixture. (Ar: argon; O2: oxygen; ICP: intracranial pressure measurement).

Utilizing the determined optimal argon concentration from test series A, we assessed the optimal duration of argon application with two sub-test series. Start of argon treatment was 30 min before CCI. 50% argon/50% oxygen were used in the therapeutic group corresponding to results of prior in-vitro experiments and to our own findings in the first test series [3]. Control animals received room air. Moreover, an oxygen control group treated with 50% oxygen/50% nitrogen was introduced (Figure 2).

#### 2.3.3. Test Series C: Treatment at Physiological Oxygen Levels

**Figure 3:**
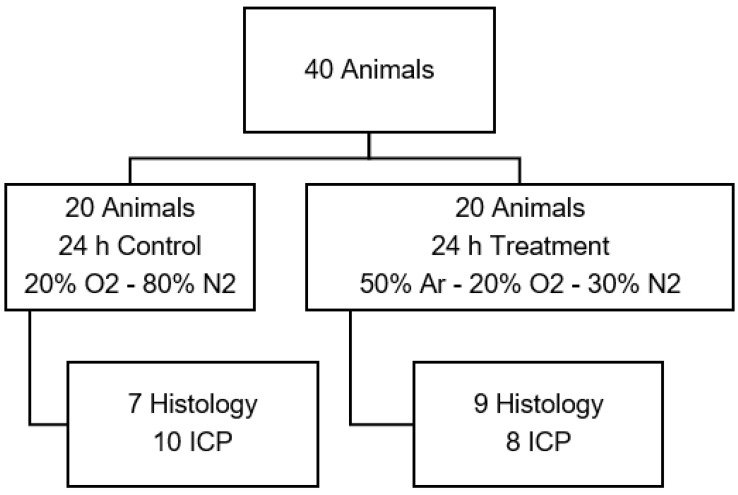
Animal distribution of test series C with physiological oxygen levels. (Ar: argon; O2: oxygen; ICP: intracranial pressure measurement).

This series was introduced to eliminate any interference that might occur because of elevated oxygen levels. Control animals received 20% oxygen and 80% nitrogen. The animals treated with argon received 20% oxygen, 50% argon, and 30% nitrogen. Start of therapy was 30 min before and 24 h after CCI in both subgroups (Figure 3).

#### 2.3.4. Test Series D: Effects of Argon on Mean Arterial Pressure and Cerebral Blood Flow after CCI

After determining the optimal concentration of argon, we analyzed mean arterial pressure (MAP) and cerebral blood flow (CBF) 30 min before, until 2 h after CCI. Argon therapy (50% Ar, 50% O_2_) or O_2_/N_2_ (50% O_2_, 50% N_2_) was administered during the whole duration of the experiment. For animal distribution and study procedures, see Section 2.7.

### 2.4. Neuroscores and Beamwalk Testing

Neurological status was assessed 24 h after trauma after a modified Dixon’s and Bederson’s neuroscore (Table 1) [15,16,17]. Mice underwent beamwalk testing, crossing a 40-cm-long non-slip beam (diameter 1.5 cm) three times; missteps of the left leg were counted during that procedure.

### 2.5. Histology and BCV

After sacrificing the animals assigned for BCV measurement (after 24 h, exception: 15-min controls) through cervical dislocation in deep isoflurane anesthesia, brains were removed and frozen at −70 °C. Coronal slices were prepared with a cryostat. Ten micrometer coronal slices were collected with an interslice thickness of 500 μm throughout the entire brain. Slices were H and E (hematoxylin and eosin)-stained and contusion areas a**_n_** for each slice were measured—n being the number of slices—using digital image analyzing software and microscope (Olympus Cell Sense, Olympus Lifescience, Hamburg, Germany). BCV was calculated using the formula

V_Contusion/_mm^3^ = (a_1_ + a_2_ + a_3_ + ⋯ + a_n_) × 0.5 mm.

**Figure 4:**
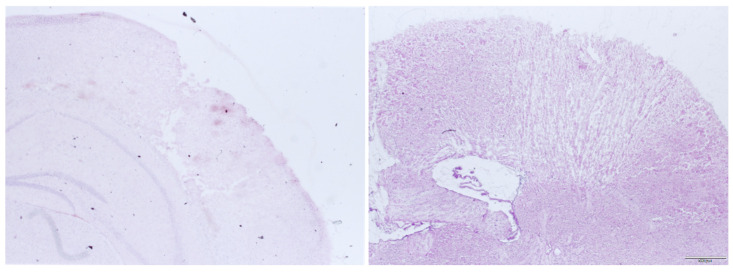
Histological preparations of the right neocortex 15 min after CCI (**left**) and 24 h after CCI (**right**).

Figure 4 shows an example of the focal lesion 15 min after trauma and 24 h after trauma. It can be seen that there is a clearly demarcated area of contusion that is readily accessible to histologic volume measurement. In other damage models like weight drop, impact acceleration or fluid percussion, there is more diffuse subcortical damage, which cannot be evaluated by the examination used here [15,16,17,18,19].

### 2.6. BWC and ICP Measurement

All animals assigned for ICP and BWC were put in short inhalative anesthesia 24 h after CCI. Contralateral to the trauma side, the temporal muscle was mobilized by blunt preparation and a small trephination was performed in the lateral temporal bone. An ICP microsensor (CP Express, Codman Neuro, Raynham, MA, USA) was inserted in the epidural space of the contralateral hemisphere after being calibrated in distilled water. After 5 min the ICP value was registered. Brains were promptly removed, cerebellum and olfactory bulbs removed and hemispheres divided. They were then measured wet (W_Wet_) and again after drying for 24 h at 90 °C (W_Dry_). BWC of the hemispheres was then determined via:

P_Water_ = (W_Wet_ − W_Dry_)/W_Wet_

### 2.7. MAP and CBF Measurement

For this study 18 mice were anesthetized with intraperitoneal injections of medetomidine, midazolam, and fentanyl. As the experiments had to last at least 2.5 h, mice were intubated and mechanically ventilated (MiniVent 845, Hugo Sachs Elektronik, March-Hugstetten, Germany). Gas concentrations were 50% oxygen and 50% nitrogen for all animals prior to CCI. In order to ensure appropriate ventilation, arterial blood gas analyses were taken at regular intervals (after 0, 30, 90, 150 min).

In order to monitor MAP the femoral arteries were dissected and a fluid filled catheter inserted into the femoral artery and the pressure measured by a manometer (Pressure Monitor BP-1, World Precision Instruments, Sarasota, FL, USA). To control the cerebral blood flow the right temporal muscle was detached from the skull with a scalpel. The laser Doppler probe (Periflux 5000, Perimed, Järfälla, Sweden) was then fixated directly on the skull. All data were automatically recorded and saved at 1 Hertz via computer interfaces (Labscribe 2, iWorx, Dover, NH, USA). CCI was performed as stated above.

MAP and cerebral blood flow were recorded for 30 min prior to CCI in order to have baseline values for all animals. After CCI, 8 control-group animals continued to receive 50% oxygen and 50% nitrogen, whereas 10 argon-treated mice received 50% argon and 50% oxygen. Treatment was performed for two hours with continuous monitoring of MAP and CBF. Afterwards the mice were sacrificed. We refrained from assessing these animals neurologically due to limited validity after long anesthesia.

### 2.8. Statistical Analysis

Statistical work was performed with SPSS 22 (IBM Corp. Released 2013. IBM SPSS Statistics for Windows, Version 22.0. Armonk, NY, USA). ANOVA with an unpaired *t*-test analysis was used for ICP, BWC, BCV, and beamwalk testing. For neuroscores a Mann–Whitney U test was used. All results are displayed as mean value ± standard deviation (SD).

## 3. Results

### 3.1. Test Series A: Optimal Argon Concentration

No animals died during treatment.

#### 3.1.1. Primary Brain Damage

BCV in the 15-min control group, representing primary damage after CCI were 10.5 ± 1.1 mm^3^. Compared with the 24 h control group (19.4 ± 3.2 mm^3^), there was a mean growth of BCV by 8.9 mm^3^ (85% of primary BCV) (*p* < 0.001) due to secondary brain damage.

#### 3.1.2. Effect of Argon on BCV

**Figure 5:**
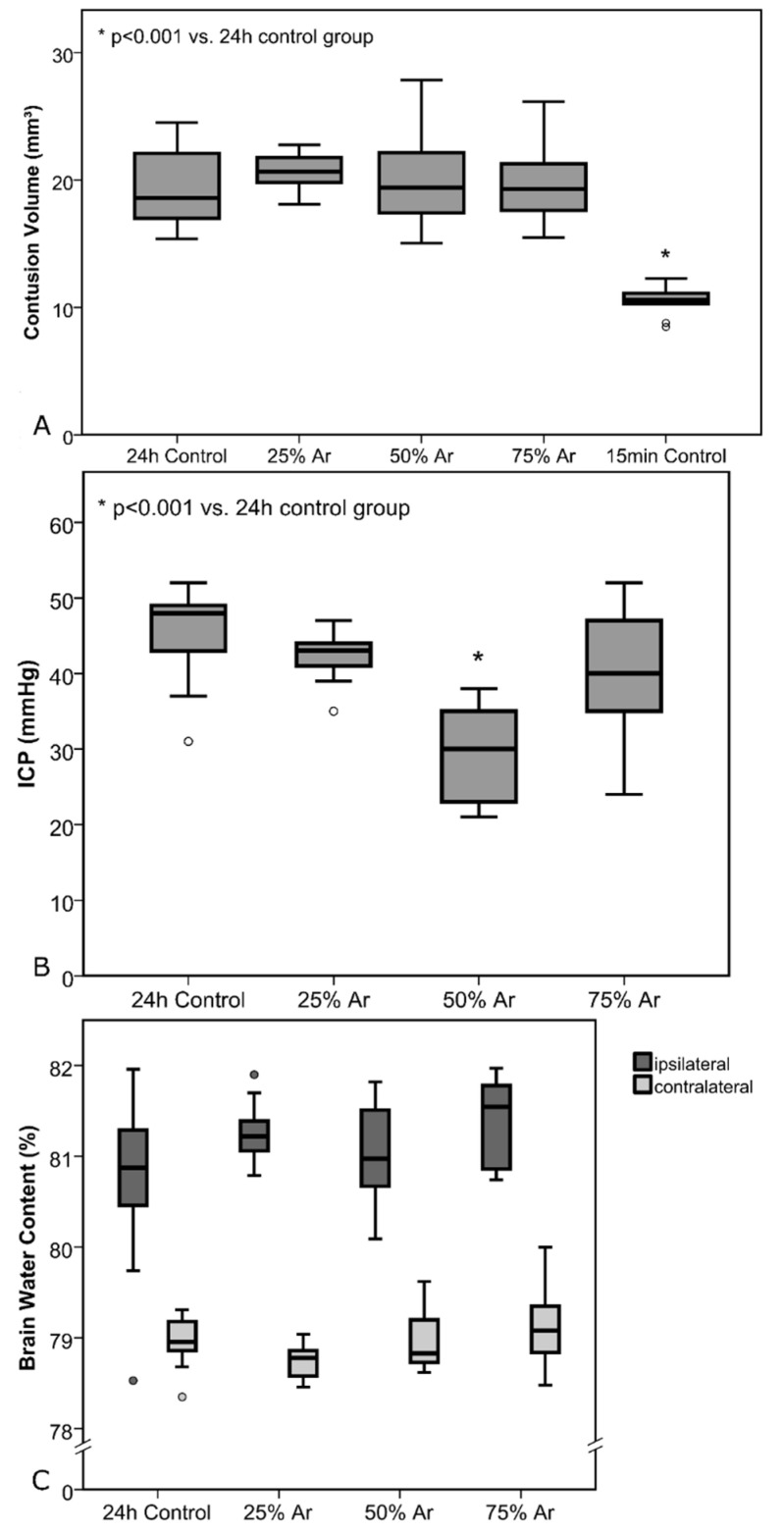
Test series A: Treatment was administered 30 min before trauma until 4 h after trauma. All results are shown as standard boxplot charts. (**A**) Contusion volumes for the control groups and the animals treated with argon. Brains for the 15 min control group were removed after 15 min to determine primary damage. All other brains were removed 24 h after CCI. There was a highly significant increase due to secondary damage when comparing 15 min and 24 h control groups (*p* < 0.001). The number of animals per group were as follows: 24 h control = 10; 25% Ar = 8; 50% Ar = 10; 75% Ar = 9; 15 min control = 10. (**B**) Intracranial pressure (ICP) values 24 h after TBI. There was a highly significant difference between the 24 h control and 50% Ar group (*p* < 0.001). (**C**) Brain water content (BWC) values of each hemisphere 24 h after TBI, divided by groups (the number of animals per group in Graph B and C were as follows: 24 h control = 9; 25% Ar = 9; 50% Ar = 10, 75% Ar = 9).

There were no significant differences in BCV in all groups (Figure 5).

#### 3.1.3. Effect of Argon on ICP and BWC

ICP directly before removal of the brains showed mean ICP values of 44.9 ± 6.4 mmHg (24-h control), 42.3 ± 3.6 mmHg (25% argon, *p* = 0.31), 29.5 ± 6.2 mmHg (50% argon, *p* < 0.001), and 39.2 ± 8.9 mmHg (75% argon, *p* = 0.13) (Figure 5).

Mean BWC in the ipsilateral hemispheres were as follows: 80.72 ± 0.98% (24 h control), 81.27 ± 0.35% (25% argon, *p* = 0.13), 81.03 ± 0.53% (50% argon, *p* = 0.40), and 81.40 ± 0.47% (75% argon, *p* = 0.65). There was significant increase in BWC due to trauma in all groups in the ipsilateral hemispheres (*p* < 0.001) compared with the contralateral hemispheres (Figure 4). Mean BWC in the contralateral hemispheres did not differ significantly (Figure 5).

#### 3.1.4. Neurological Outcome and Beamwalk Test

**Figure 6:**
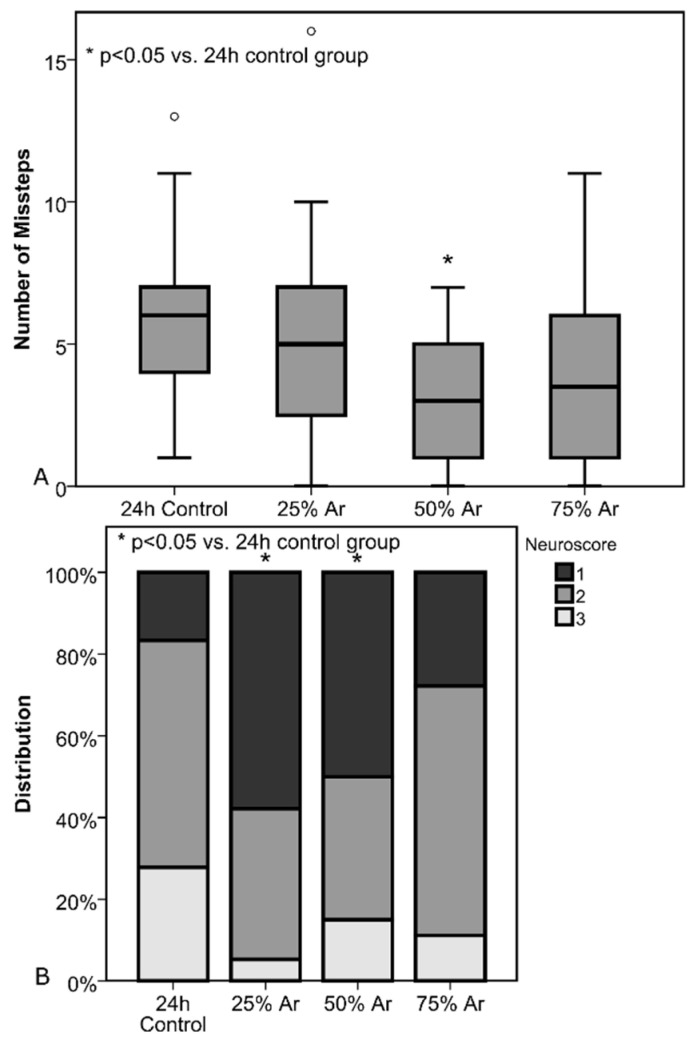
Treatment was administered 30 min before trauma until 4 h after trauma (the number of animals were as follows: 24 h control = 19; 25% Ar = 17; 50% Ar = 20; 75% Ar = 18) (**A**) total number of beamwalk missteps 24 h after trauma. There was a significant decrease in missteps when comparing the 50% Ar group to control. Results are shown as standard boxplot charts. (**B**) Animal distribution according to Dixon’s and Bederson’s neuroscores 24 h after trauma. There was a significant improvement when comparing the 25% and 50% Ar group to control.

Beamwalk was assessed 24 h after CCI for all animals except the 15-min control. Cumulative numbers of missteps in three walks were 5.8 ± 3.1 (24 h control), 5.2 ± 3.8 (25% argon, *p* = 0.56), 2.8 ± 2.2 (50% argon, *p* = 0.001), and 4.2 ± 3.3 (75% argon, *p* = 0.14). There was a significant reduction of missteps in the group treated with 50% argon (*p* = 0.001) compared with the control group (Figure 6).

Neuroscores were obtained at the same time as the beamwalking test. The scores were 2.1 ± 0.7 (24 h control), 1.47 ± 0.6 (25% argon, *p* = 0.01), 1.7± 0.8 (50% argon, *p* = 0.049), and 1.8 ± 0.6 (75% argon, *p* = 0.21). There was a significant improvement in neuroscores in animals treated with 25% (*p* = 0.01) and 50% argon (*p* = 0.049) when compared with controls (Figure 6).

### 3.2. Test Series B: Optimal Duration of Treatment

Each group contained 19 animals for analysis. One animal of the room air control group and one of the argon-treated group died during treatment, approximately 30 min and 6 h after trauma, respectively. One animal of the oxygen control group died before trauma during preparation of the craniotomy.

#### 3.2.1. Effect of Argon on BCV

BCV was determined as above mentioned after 24 h. There were three groups. Mean BCV were 21.6 ± 2.4 mm^3^ (room air control), 21.7 ± 4.0 mm^3^ (control group, oxygen), and 19.7 ± 3.4 mm^3^ (argon treatment), respectively (n.s.).

#### 3.2.2. Effect of Argon on ICP and BWC

ICP measurements after 24 h were as follows: 45.4 ± 9.3 mmHg (room air control group), 47.4 ± 5.7 mmHg (oxygen control group), and 42.4 ± 4.4 mmHg (argon treatment) (n.s.). BWC in the ipsilateral hemispheres after 24 h showed percentages of 81.0 ± 0.7% (room air control group), 81.1 ± 0.4% (oxygen control group), and 80.8 ± 0.6% (argon treatment) (n.s.). BWC in the contralateral hemispheres were 79.0 ± 0.4% (room air control group), 78.9 ± 0.4% (oxygen control group), and 78.8 ± 0.4% (argon treatment), respectively (n.s.). There was a highly significant (*p* < 0.001) difference in BWC when comparing ipsilateral and contralateral hemispheres in all groups.

#### 3.2.3. Neurological Outcome and Beamwalk Test

The number of missteps in beamwalk tests did not show any significant difference when comparing argon treatment (3.0 ± 4.3) with room air control (4.0 ± 2.2) (*p* = 0.36) and oxygen control (5.16 ± 2.9) (*p* = 0.08). The neuroscores of argon treated mice (1.7 ± 0.6) improved significantly when compared with oxygen control animals (2.5 ± 0.7) (*p* = 0.001), while there was no significant change when compared to room air controls (1.9 ± 0.7) (*p* = 0.37).

### 3.3. Test Series C: Treatment at Physiological Oxygen Levels

The introduction of an oxygen control group showed worse outcomes for animals that received 50% oxygen and 50% nitrogen. Although not significant, the difference between oxygen control and argon treatment prompted us to introduce a test series in which we set oxygen at 20%. Seventeen animals were included in the analysis in each group. Six subjects were excluded due to methodical reasons of failed recording. No animal died.

#### 3.3.1. Effect of Argon on BCV

There was no significant difference in mean BCV after 24 h when comparing argon treated mice (25.6 ± 3.1 mm^3^) with control mice (27.3 ± 1.5 mm^3^) (*p* = 0.19).

#### 3.3.2. Effect of Argon on ICP and BWC

There was no significant difference in mean ICP after 24 h when comparing argon-treated animals (43.7 ± 8.3 mmHg) with the control group (47.6 ± 8.0 mmHg) (*p* = 0.30). In the ipsilateral hemispheres BWC after 24 h was 81.3 ± 0.5% for argon treatment and 81.3 ± 0.4% for controls (n.s.) BWC in the contralateral hemispheres showed no significant difference between argon treatment (79.0 ± 0.4%) and control (79.0 ± 0.3%) (*p* = 0.78).

There was a highly significant (*p* < 0.001) difference between ipsilateral and contralateral hemispheres in both groups in BWC.

#### 3.3.3. Neurological Outcome and Beamwalk Test

There was no significant difference when comparing neuroscores of argon treatment (2.4 ± 0.8) and control (2.1 ± 0.9) (*p* = 0.40). Moreover, there was no significant difference between argon treatment (6.2 ± 3.6) and control (5.3 ± 3.2) (*p* = 0.41).

### 3.4. Test Series D: Effects of Argon on MAP and CBF after CCI

**Figure 7:**
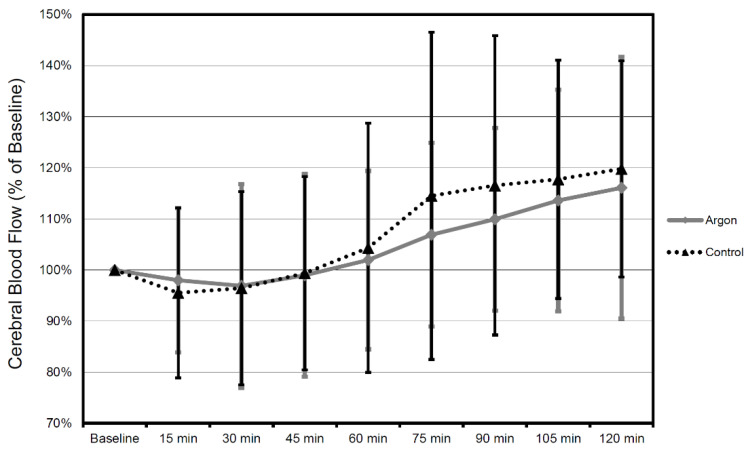
CBF values in time course 2 h after trauma in 15 min intervals. All values in relation to baseline 30 min prior to trauma (mean ± SD). Therapy was administered until 2 h after trauma (the number of animals were as follows: Argon = 9, Control = 9).

**Figure 8:**
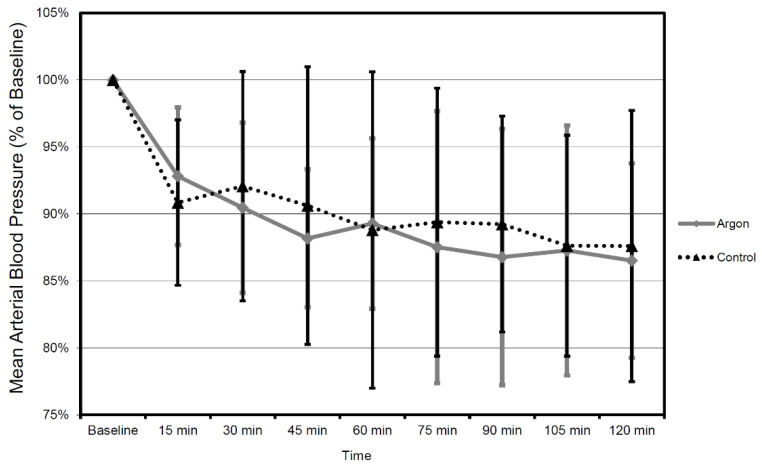
Mean arterial blood pressure (MAP) values in time course 2 h after trauma in 15 min intervals. All values in relation to baseline 30 min prior to trauma (mean ± SD). Therapy was administered until 2 h after trauma (the number of animals were as follows: Argon = 9, Control = 9).

When comparing the mean values between argon treatment and controls, there was no significant difference in mean CBF (all *p*-values at *p* = 0.53 or above) and MAP (all *p* values at *p* = 0.46 or above) during each interval for the entire recorded period of time (Figure 7 and Figure 8).

## 4. Discussion

The results suggest a certain neuroprotective effect, namely at a functional level and at lowering ICP. However, these results have to be considered with caution.

There has been no significant reduction of BCV. If at all, we could observe a slight increase of BCV in all groups treated with argon compared to controls. Moreover, BWC of both hemispheres were at almost identical levels in all groups. Somewhat contradictory to these results is the observed reduction of ICP in mice treated with 50% argon. Likewise, the improved functional outcome in mice treated with 25% and 50% argon seem to have to no correlate in the other findings. We suspect that an inhibition of apoptosis leads to an increased number of vital neurons within the contused tissue and therefore to a better outcome. This could be shown in Loetscher’s in-vitro model of TBI and OGD where propidium iodide staining was used to identify necrotic cells [3]. The most effective concentration was 50% in TBI. However, slices were incubated for 72 h as opposed to the 4 h we used. A similar study has also shown in-vitro neuroprotection of argon in OGD after 24 h of incubation [20].

Several studies suggest a mechanism of action not involving ion channels. Argon is capable of inducing heat shock proteins via activation of ERK1/2, increasing Bcl-2 expression, increasing expression of inflammatory markers, inhibiting apoptotic proteins like caspase-3, and modifying tPA activity [7,8,9,10,21,22,23,24].

The ERK1/2 pathway is essential in the regulation of the cell cycle and apoptosis. It has significant importance in oncological studies, as defects might lead to uncontrolled activation and therefore uncontrolled growth of tumors. A study by Ulbrich et al. demonstrated increased expression of extracellular-signal-regulated kinase 1/2 (ERK1/2) and consecutive reduction of heme oxygenase-1 (HO-1) in rat retinal ganglion cells in vivo. For this purpose, 75% argon and 25% oxygen were administered to rats for 1 h after 60-min ischemia and reperfusion of one eye [8].

The antiapoptotic and neuroprotective effect by argon shown via ERK1/2 itself is mediated by decrease of density and expression of TLR (toll-like-receptor) 2 and 4 after rotenone-induced cell damage. Moreover, argon’s effect can be attenuated by administering inhibitors of the ERK1/2 and TLR pathway [25,26].

In an in-vivo models of stroke, i.e., middle cerebral artery occlusion (MCAO), 50% argon proved to reduce total infarct volume and functional outcome after 2 h of ischemic trauma in rats [5]. In this study, rats were administered 50% argon and 50% oxygen via facemask for 1 h after middle cerebral artery occlusion, i.e., intraischemic. In this context, the effect of noble gases on thrombolysis is of importance. Both argon and xenon appear to have dose-dependent inhibitory and excitatory effects on tPA (tissue plasminogen activator) activity. This is of particular relevance to ischemia-perfusion models (i.e., MCAO models) [27]. Argon inhibited the activity of tPA at a concentration of 25% in the study by David et al., while a high concentration of 75% promoted activity. The concentration of 50% used here had almost no effect on tPA [9].

In contrast, a study exists in which an improvement of the neurological status and additionally a reduction of the infarct area was observed after 2 h MCAO and argon treatment for 60 min in rats. However, the number of vital cells in the penumbra did not differ significantly from control. In addition, expression analysis revealed significantly increased expression of both pro-inflammatory mediators (IL-1β, IL-6, iNOS) and neuroprotective factors [10]. Another study found neuroprotective properties of argon when given in vitro after OGD [4]. The same study has shown neuroprotection after intra-striatal injection of NMDA in vivo in rats.

NMDA receptors are an important mediator in the development of secondary brain damage, leading to higher intracellular ion influx after massive glutamate release due to widespread depolarization of neurons and consecutive cellular swelling and cell death, hence excitotoxicity [28,29]. Xenon, another noble gas, has already shown neuroprotection by modifying the glycin-receptor site of NMDA-channels. However, argon does not seem to work through the same mechanism of action [30]. ATP-dependent potassium channels have also been ruled out in another in-vivo study [31]. A potentiation of GABA receptors due to argon is under discussion [32].

Regarding craniocerebral trauma in general, in-vivo studies have investigated the mechanisms of impaired cell function and apoptosis extensively. In-vivo CCI in juvenile rats leads to upregulation of binding immunoglobulin protein (BiP) and C/EBP homologous protein (CHOP) as early as 4 h. After 72 h, there was increased expression of hypoxia-inducible factor (HIF)-1α. In the first case, this indicates an increased load on the endoplasmic reticulum, in the second case, a delayed onset of tissue hypoxia [33]. After an in-vivo blast-type TBI in rats, there is diffuse microvasculature damage and axonal injury with reactive gliosis, in the absence of macroscopic lesions such as brain edema. Impaired calcium homeostasis leads to activation of calpain and increased degradation of spectrin, resulting in impaired axonal integrity and synaptic function [34].

In context, this study has limitations. No effect of argon could be shown in this work with respect to the size of the contusion volume. The aforementioned in-vitro studies showed a reduction in damage due to propidium iodide staining, which is more indicative of cell viability than contusion volume. The HE staining performed in this study was able to detect the boundaries of the contusion area and thus the volume of contusion. The survival of the cells in the contusion volume itself cannot be reliably assessed by this stain, which could explain the discrepancy between better functional outcome and unchanged contusion volume. Further immunohistochemical studies can more precisely investigate the effect of argon on apoptotic and necrotic signaling pathways, possibly in deeper brain structures that appear to be without pathological findings in HE staining. Yet, data on the potential effects of argon and other noble gases on TBI is still rare. Xenon, as another noble gas, reduces the neurotoxic and proapoptotic effects of propofol, for instance [35]. In a model of myocardial infraction, argon proved to be protective against apoptosis due to ischemia via activation of Erk, Akt, but also a biphasic regulation of JNK [36]. The same reduction of ischmia-related apoptosis is true for helium as another noble gas [37].

Summarizing all findings of this and former studies, we suspect neuroprotective properties of argon when administered in vivo after CCI in mice through inhibition of apoptosis. However, the lack of reduction of BCV and BWC require further studies. This does particularly involve the extension of treatment durations, as argon was applied for 72 h in the only study that has shown neuroprotection after (in vitro) TBI. Additionally, as latest research suggests that argon does not target ion channels but modifies gene expression, a longer treatment duration might enhance these mechanisms and improve results. If the ERK1/2 pathway is indeed the main course of action supplementary drug intervention may further facilitate and enhance antiapoptotic effects [10,25,30,38].

## 5. Conclusions

In summary, argon exerts neuroprotection mainly regarding functional outcome and to a lesser extent ICP. There is no effect on BCV, BWC, MAP, or CBF. Being efficient in improving functional outcome, argon has the potential to be used as an additional option besides established anti-edematous therapies. However, it has to be taken into consideration that the control group in the first test series received different oxygen levels, weakening the informative value of the significant results in this series. More experiments regarding the involved molecular pathways are needed to confirm the inhibition of apoptosis and the underlying mechanisms thereof.

## Figures and Tables

**Table 1:** A modified Dixon’s and Bederson’s score was used to evaluate neurological functionality 24 h after CCI. The sum of all subcategories yielded the final score.

Subcategory	Subcategory Score
1. spontaneous activity:	0 = active, targeted search 1 = active, uncontrolled 2 = reduced 3 = none
2. apnoea/seizure:	0 = none 1 = once 2 = twice 3 = frequently
3. corneal reflex:	0 = yes 1 = no
4. paw flexion:	0 = yes 1 = no
5. head control:	0 = safe 1 = weak 2 = none
6. startle reflex:	0 = yes 1 = no
7. rising from supine positon:	0 = cannot be put in supine position 1 = rises from supine position spontaneously 2 = rises from supine position with assistance 3 = rising not possible
8. balancing:	0 = safe 1 = unsafe 2 = walking on beam not possible 3 = sitting on beam not possible
9. positional reflex:	0 = yes 1 = no
10. lateral pressure resistance:	0 = present 1 = reduced
11. circling:	0 = no 1 = against resistance 2 = spontaneous

## Data Availability

The data that support the findings of this study are available from the corresponding author, S.M.K., upon request.
